# *PredPlantPTS1*: A Web Server for the Prediction of Plant Peroxisomal Proteins

**DOI:** 10.3389/fpls.2012.00194

**Published:** 2012-08-27

**Authors:** Sigrun Reumann, Daniela Buchwald, Thomas Lingner

**Affiliations:** ^1^Center for Organelle Research, University of StavangerStavanger, Norway; ^2^Department of Bioinformatics, University of GöttingenGöttingen, Germany

**Keywords:** PTS1, peroxisome, machine learning, *Arabidopsis*, orthologs, subcellular targeting, proteome

## Abstract

Prediction of subcellular protein localization is essential to correctly assign unknown proteins to cell organelle-specific protein networks and to ultimately determine protein function. For metazoa, several computational approaches have been developed in the past decade to predict peroxisomal proteins carrying the peroxisome targeting signal type 1 (PTS1). However, plant-specific PTS1 protein prediction methods have been lacking up to now, and pre-existing methods generally were incapable of correctly predicting low-abundance plant proteins possessing non-canonical PTS1 patterns. Recently, we presented a machine learning approach that is able to predict PTS1 proteins for higher plants (spermatophytes) with high accuracy and which can correctly identify unknown targeting patterns, i.e., novel PTS1 tripeptides and tripeptide residues. Here we describe the first plant-specific web server *PredPlantPTS1* for the prediction of plant PTS1 proteins using the above-mentioned underlying models. The server allows the submission of protein sequences from diverse spermatophytes and also performs well for mosses and algae. The easy-to-use web interface provides detailed output in terms of (i) the peroxisomal targeting probability of the given sequence, (ii) information whether a particular non-canonical PTS1 tripeptide has already been experimentally verified, and (iii) the prediction scores for the single C-terminal 14 amino acid residues. The latter allows identification of predicted residues that inhibit peroxisome targeting and which can be optimized using site-directed mutagenesis to raise the peroxisome targeting efficiency. The prediction server will be instrumental in identifying low-abundance and stress-inducible peroxisomal proteins and defining the entire peroxisomal proteome of *Arabidopsis* and agronomically important crop plants. *PredPlantPTS1* is freely accessible at ppp.gobics.de.

## Introduction

For most eukaryotic organisms whose genome has been sequenced, the majority of encoded proteins have remained of unknown function and subcellular localization. Identifying the complete proteome of cell organelles by experimental methodologies represents a challenging task, particularly for small and fragile organelles such as peroxisomes (Reumann et al., 2007, 2009; Eubel et al., 2008; for review see Reumann, 2011). In the post-genomic era, computational tools for the prediction of subcellular targeting of nuclear-encoded proteins have become indispensable to correctly assign unknown proteins to compartment-specific protein networks and to ultimately determine protein function (Nair and Rost, 2004; Schneider and Fechner, 2004; Mintz-Oron et al., 2009).

Peroxisomes are small, ubiquitous eukaryotic organelles that are highly complex, and dynamic in functions and mediate a wide range of oxidative metabolic activities. Plant peroxisomes are essential for lipid metabolism, photorespiration, and hormone metabolism, and they play pivotal roles in plant responses to abiotic and biotic stresses (Lopez-Huertas et al., 2000; Hayashi and Nishimura, 2003; Lipka et al., 2005; Nyathi and Baker, 2006; Reumann and Weber, 2006; for review see Kaur et al., 2009; Hu et al., 2012).

Contrary to mitochondria and plastids, peroxisomes completely lack any residual genome and transcription machinery. Thus, all peroxisomal matrix proteins have to be imported from the cytosol (Purdue and Lazarow, 2001). Apart from a few exceptions, proteins are targeted to the peroxisome matrix by a conserved peroxisome targeting signal of either type 1 (PTS1) or type 2 (PTS2). The PTS1 comprises the C-terminal domain of ~10–15 amino acids (aa) and is often largely determined by C-terminal PTS1 tripeptides such as SKL> or SRM> (where “>” indicates the C-terminal end of the protein). The PTS2 is commonly represented by a conserved nonapeptide of the prototype RLx_5_HL located in the N-terminal protein domain comprising approximately 50 aa (Reumann, 2004).

Prediction methods have been previously developed, mainly for metazoa, to predict PTS1 proteins from genomic sequences (Emanuelsson et al., 2003; Neuberger et al., 2003a,b; Boden and Hawkins, 2005; Hawkins et al., 2007). However, plant-specific PTS1 protein prediction methods had long been lacking. Moreover, previous PTS1 protein prediction models were not designed to infer novel PTS1 tripeptides or predict low-abundance proteins (Emanuelsson et al., 2003; Neuberger et al., 2003b; Boden and Hawkins, 2005; Hawkins et al., 2007). Recently, we presented a discriminative machine learning approach to the prediction of plant peroxisomal PTS1 proteins (Lingner et al., 2011). The two different algorithms applied showed high prediction accuracy and were able to correctly predict novel PTS1 tripeptides including formerly unknown tripeptide residues. While the simpler PWM (position weight matrix) model demonstrated a high sensitivity and predicted >380 *Arabidopsis* PTS1 proteins, the more complex RI (residue interdependence) model emerged as too stringent for the prediction of PTS1 proteins and detection of novel PTS1s.

In order to make PTS1 prediction methods practically applicable, several online resources have been presented (Emanuelsson et al., 2003; Neuberger et al., 2003b; Boden and Hawkins, 2005; Schluter et al., 2010). These web servers consistently allow the upload of one or more sequence(s) for evaluation with the corresponding prediction method and provide prediction output in terms of the information whether the protein is likely to be targeted to peroxisomes and/or a value indicating the targeting probability. However, none of these resources so far allows assessing the importance of particular amino acids within the C-terminal region with respect to peroxisome targeting. Such information might be useful for experimental researchers to increase the peroxisome targeting efficiency of weakly targeted cargo by site-directed mutagenesis. Furthermore, for sequences with non-canonical PTS1 tripeptides, pre-existing web servers do not inform the user whether the C-terminal tripeptide has been experimentally verified before as a PTS1 tripeptide.

Here, we present *PredPlantPTS1*, a web server for the prediction of plant peroxisomal proteins carrying a PTS1. *PredPlantPTS1* provides an easy-to-use web interface for sequence submission, an interpretable output in terms of total and residue-specific prediction scores, and PTS1 tripeptide evaluation.

## Results and Discussion

### Web interface

The *PredPlantPTS1* server is implemented in PHP (user interface) and Perl (prediction engine) and is freely accessible at ppp.gobics.de. In particular, the web server does not require a login or the specification of an email address. The submission page of *PredPlantPTS1* allows the user to provide a single protein sequence in FASTA or plain text format. Invalid sequence characters such as numbers, white spaces, and special symbols are stripped off automatically, allowing direct use of protein sequences, for instance, from GenBank and TAIR protein information files (www.arabidopsis.org). For each submission a unique job-ID is generated and stored for future access. The underlying prediction algorithm is that of the more sensitive PWM model, which uses the C-terminal 14 aa to predict peroxisome targeting (Lingner et al., 2011).

As a demonstration example for the following analyses, the *Arabidopsis* gene model At1g18700 is used, which is of yet unknown function and is annotated as DNAJ heat shock N-terminal domain-containing protein by TAIR10. By alternative splicing the gene is expressed in four protein variants differing in length between 695 and 715 aa residues. Three variants share the same C-terminal 14 aa domain (KDAVQILSSGSDSD>, At1g18700.1/3/4), while the second variant terminates with the PTS1-related tripeptide, QRL> (ILSSVRSMKGFQRL>). Neither QRL> nor Gln at position −3 have been experimentally validated as a plant PTS1 tripeptide or PTS1 tripeptide residue, respectively (Lingner et al., 2011), necessitating the application of computational methods to predict peroxisome targeting.

#### Position-specific prediction scores

**Figure 1:**
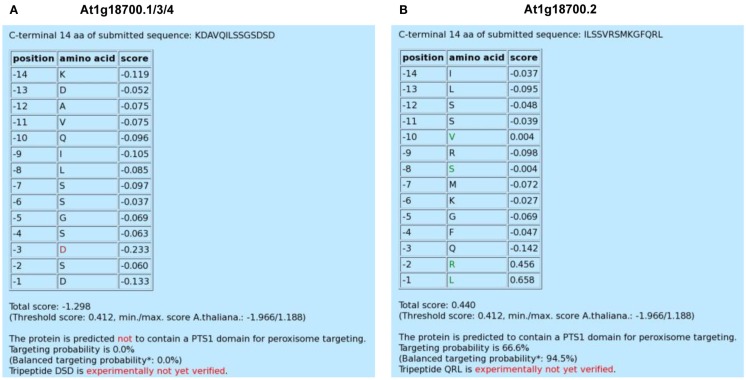
**Screenshot of the *PredPlantPTS1* result page for three alternative splice variants (At1g18700.1/3/4) of an unknown DNAJ heat shock N-terminal domain-containing protein carrying the same C-terminal domain (A) and the specific variant, At1g18700.2, which terminates with the putatively novel PTS1 tripeptide, QRL>**. **(B)** All four variants differ in size (At1g18700.1, 700 aa; At1g18700.2, 705 aa; At1g18700.3, 695 aa; At1g18700.4, 715 aa). Residues with PWM scores that lie outside a defined interval are highlighted in green and red colors, respectively, and indicate a predicted positive and negative effect on peroxisome targeting by the PTS1 pathway, respectively.

**Figure 2:**
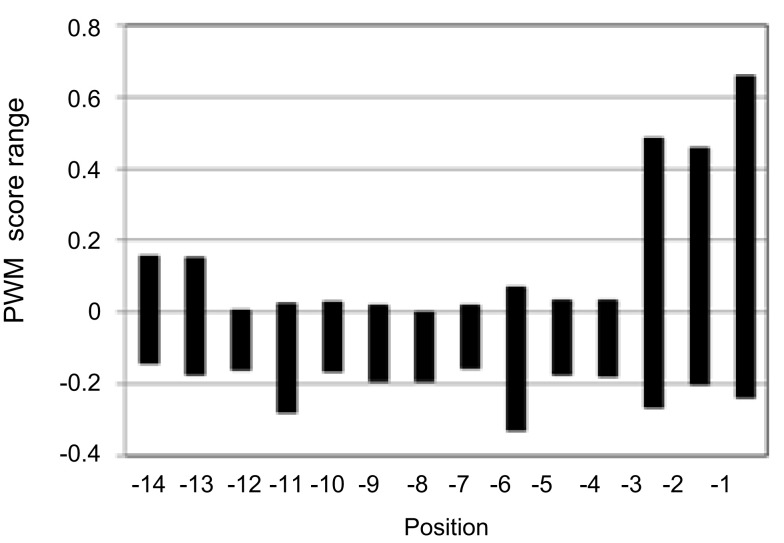
**Position-specific prediction score range of the general PWM score matrix of plant PTS1 proteins**. From the matrix values of each amino acid residue the position-specific range of values has been determined and the mean value (−0.069) and the standard deviation have been calculated separately for the PTS1 tripeptide (0.112) and the 11 upstream residues (0.057) to color extreme aa or amino acid residues of high (green) and low (red) PWM prediction scores on the result page of *PredPlantPTS1*.

The prediction generally takes less than a second. After activating the “Predict” button, the user is instantly directed to the *PredPlantPTS1* result page associated with a particular job (Figure 1). Here, a list of the 14 C-terminal amino acids of the submitted sequence is shown along with the predicted position-specific scores. Such a position-specific score indicates whether a particular residue at a particular sequence position is predicted to enhance (more positive score) or reduce (more negative score) peroxisome targeting. The position-specific range of PWM scores of all 20 possible aa residues illustrates that the three C-terminal tripeptide residues determine predicted peroxisome targeting to maximum degree, followed by position −6 and −11 (Figure 2).

By calculating the mean value (−0.069) and standard deviation (SD) of all position-specific scores separately for the C-terminal tripeptide (0.112) and the 11 upstream residues (0.057), we defined an upper (mean + SD) and lower threshold (mean − SD) for the identification of position-specific residues that are predicted to be highly important for peroxisome targeting. Scores that lie outside the interval defined by these thresholds are highlighted in green and red colors, respectively, and indicate a predicted positive and negative effect on peroxisome targeting by the PTS1 pathway, respectively. For instance, the red Asp residue at position −3 of At1g18700.1/3/4 indicates that particularly this acidic residue lowers the targeting probability of the full-length protein (Figure 1A). On the other hand, four residues of At1g18700.2 (L, position −1; R, position −2; S, position −8; V, position −10) are highlighted in green and are predicted to be most decisive to enhance peroxisome targeting by the non-canonical putatively novel PTS1 tripeptide, QRL> (Figure 1B).

#### Total prediction score and its interpretation by posterior probabilities

Below this residue-specific list, the total prediction score is provided, which represents the sum of the 14 position-specific PWM scores for the analyzed sequence of interest (Figure 1). Additionally, the threshold of the total prediction score (0.412) is given, which has been deduced from >2,500 plant PTS1 protein example sequences derived from approximately 260 plant species and is universal to Spermatophytes (Lingner et al., 2011). To integrate the absolute prediction scores in the context of the entire score range, the maximum (1.188) and minimum (−1.966) prediction scores are provided in parentheses representatively for the model organism *Arabidopsis* (Lingner et al., 2011). If the total prediction score is below the threshold (e.g., −1.298 for At1g18700.1/3/4), the given sequence is predicted not to contain a PTS1 domain for peroxisome targeting. By contrast, if the score is equal to or exceeds the threshold (e.g., 0.440 for At1g18700.2), the given sequence is predicted to contain a functional PTS1 domain (Figures 1A,B).

In Lingner et al. (2011) we described how the algorithms were calibrated to provide targeting posterior probability values associated with the prediction scores. On the result page of *PredPlantPTS1*, two targeting probability values are shown: first, the posterior probability value calibrated as described in the original work (Lingner et al., 2011). Second, a balanced probability value based on a different calibration is shown in parentheses. Here, the posterior probability values have been calibrated by assuming an equal variance of positive (PTS1) and negative (non-PTS1) example sequence scores. The assumption of equal variance leads to a broader intermediate probability value range and higher targeting probability values for sequences differing from the majority of positive examples, i.e., non-canonical and low-abundance peroxisomal proteins. On the downside of increased sensitivity, and as a note of caution, the fraction of non-peroxisomal proteins with probability values >50% increases substantially and leads to a higher proportion of false positive predictions. For the DNAJ heat shock protein, both posterior probabilities are 0% for At1g18700.1/3/4, identifying the protein unquestionably as a non-PTS1 protein, while the second splice variant At1g18700.2 is predicted to be peroxisome-targeted by the PTS1 pathway by both the original (66.6%) and balanced (94.5%) posterior probability.

#### C-terminal tripeptide evaluation

One major property of the PWM prediction model is its capability to correctly predict unknown proteins carrying novel non-canonical PTS1 tripeptides as peroxisome-targeted. By combining the *Arabidopsis* PTS1 protein predictions with large-scale *in vivo* subcellular targeting analyses, we established 23 newly predicted PTS1 tripeptides for plants and identified several previously unknown *Arabidopsis* PTS1 proteins (Lingner et al., 2011).

Nevertheless, the correct prediction of plant proteins carrying novel non-canonical PTS1 tripeptides remains highly challenging. A few true positive plant PTS1 proteins are given prediction scores below threshold, and a few peroxisome predicted proteins could not yet be experimentally validated as peroxisomal and might represent false predictions (Lingner et al., 2011). Notably, only a small percentage of plant proteins terminating with non-canonical PTS1 tripeptides is peroxisomal because peroxisome targeting by non-canonical PTS1 tripeptides essentially depends on targeting enhancing elements located upstream of the PTS1 tripeptide, and these elements are only present in a few specific proteins. The major reason for this imperfect prediction accuracy for non-canonical PTS1 proteins is the bias of the underlying dataset of positive example sequences. Even though the sequence number is exceptionally high (>2,500 sequences) and the sequences are relatively diverse, the data set remained dominated by high-abundance proteins carrying canonical PTS1 tripeptides, most of which lack targeting enhancing patterns in the upstream domain.

As a result, the prediction of unknown proteins as being peroxisome-targeted by novel PTS1 tripeptides should be interpreted with greater caution as compared to experimentally validated PTS1 tripeptides, particularly if one of the three predicted tripeptide residues is novel. Therefore, at the bottom of the prediction result page, the user is informed whether the tripeptide of the submitted protein sequence of interest has already been verified experimentally as a functional plant PTS1 tripeptide. Such experimental validations have generally been performed by extending a fluorescent reporter protein C-terminally by the C-terminal 10 aa residues of one example *Arabidopsis* protein (Ma and Reumann, 2008; Babujee et al., 2010). For the DNAJ heat shock example protein, the user is informed that none of the two C-terminal tripeptides of either At1g18700.1/3/4 (all DSD>) or At1g18700.2 (QRL>) have been experimentally verified as plant PTS1 tripeptides, according to published literature. Our recent experimental analyses, however, confirmed that the C-terminal 10 aa residues of At1g18700.2 indeed direct enhanced yellow fluorescent protein to peroxisomes in onion epidermal cells, demonstrating that QRL> is a novel plant PTS1 tripeptide and Gln a novel residue at position −3 of the plant PTS1 motif (Chowdhary et al., 2012). The summary list of validated plant PTS1 tripeptides is frequently updated on the basis of in-house experiments, database, and literature research, and can be downloaded from the web server (Table A1 in Appendix).

### Comparison to other PTS1 protein prediction servers

*PredPlantPTS1* is the first plant-specific prediction server for PTS1 proteins. However, metazoan-specific or general online resources can be used to evaluate novel sequences with respect to predicted peroxisome targeting. For instance, the PTS1 predictor does not provide a plant-specific model, but allows running predictions according to a “general” model, which includes animals, fungi, and plants (Neuberger et al., 2003b). Furthermore, Protein Prowler can be used to analyze putative PTS1 sequences with respect to their subcellular localization (Boden and Hawkins, 2005). Here, the PTS1 detection is realized by PTS1Prowler integrated into Protein Prowler. The PeroxiP server provides a plant model for predicting PTS1 proteins but is frequently unavailable (Emanuelsson et al., 2003). As an alternative to the above-mentioned prediction servers, the PeroxisomeDB database provides peroxisome targeting prediction by means of an alignment of the C-terminus of the query sequence to previously identified conserved regions of PTS1 sequences from plants, animals, and fungi (Schluter et al., 2010).

We compared the predictions of *PredPlantPTS1* and the above-mentioned online resources using experimentally verified *Arabidopsis* proteins carrying non-canonical PTS1 tripeptides (Table 1, see also Lingner et al., 2011). Accordingly, *PredPlantPTS1* (6/10 correctly predicted peroxisomal proteins) and the PTS1 predictor (4/10) provide a good prediction sensitivity for these challenging proteins. The PTS1Prowler emerged as too restrictive and predicted none out of 10 verified PTS1 proteins correctly (50% probability threshold). The alignment-based prediction integrated within the PeroxisomeDB predicted all 10 tested sequences as peroxisomal. The reason for this is that all alignment comparisons result in an *E*-value below 10, which is the default cutoff value for the BLOCKS server. However, many non-peroxisomal proteins yield *E*-values below 10, which results in a very low prediction specificity using this cutoff value (data not shown). Lowering the *E*-value threshold may improve the prediction specificity; however, such a threshold is not provided by PeroxisomeDB. Note that we excluded PeroxiP from the evaluation, because the corresponding web server was not available during our analysis.

**Table 1:** **Comparative PTS1 protein prediction of experimentally validated *Arabidopsis* proteins carrying non-canonical PTS1 tripeptides by different web servers**.

AGI code	Acronym	C-terminal 14 aa	Exp. targ.	*PredPlantPTS1*			PTS1Prowler		PTS1 predictor		PeroxisomeDB*E*-value
				Score	Prob.	Prediction	Probability	Prediction	Score	Prediction	
At1g51745.1/2	Tudor	EARSRQQRRQRKRL>	PTD	0.615	0.990	Peroxisomal	0.00	Non-perox.	−4.86	Twilight zone	1.000
At3g01980.1/3/4	SDRc	GAQSLTRPRLKSYM>	PTD/FLP	0.610	0.989	Peroxisomal	0.00	Non-perox.	2.96	Peroxisomal	0.290
At4g16340.1	SPK1	AELSHYIPAILSEL>	PTD	0.567	0.973	Peroxisomal	0.00	Non-perox.	−27.15	Non-perox.	0.220
At1g43770.2	PHD	YLWGVFKPRQTSRY>	PTD	0.499	0.891	Peroxisomal	0.00	Non-perox.	0.73	Peroxisomal	1.000
At3g44830.1	LCAT	SDVMRMSERISIKL>	PTD	0.438	0.657	Peroxisomal	0.00	Non-perox.	−11.71	Non-perox.	1.000
At5g28360.1	ACS31	YREKENYLRLVSPL>	PTD	0.426	0.582	Peroxisomal	0.00	Non-perox.	−14.79	Non-perox.	0.210
At5g20070.1	NUDT19	VHSKQQAGVSLSSL>	FLP	0.385	0.328	Non-perox.	0.00	Non-perox.	4.275	Peroxisomal	1.000
At5g04870.1	CPK1	KMGLEKSFSIALKL>	PTD	0.321	0.080	Non-perox.	0.00	Non-perox.	−5.10	Twilight zone	0.058
At1g49350.1	pxPfkB	YNGAKMLMVHQSML>	FLP	0.298	0.044	Non-perox.	0.00	Non-perox.	−8.143	Twilight zone	0.160
At2g01880.1	PAP7	VLHRSSLSKRSAHL>	PTD	0.130	0.000	Non-perox.	0.43	Non-perox.	7.97	Peroxisomal	0.270

*The table shows prediction results of different PTS1 prediction servers (see COMPARISON TO OTHER PTS1 PROTEIN PREDICTION SERVERS) for 10 experimentally verified peroxisomal proteins from *Arabidopsis thaliana*. The full-length *Arabidopsis* sequences were submitted to the corresponding websites in April 2012. The fourth column indicates the experimental targeting result either for 10-aa peroxisome targeting domain (PTD) constructs or full-length proteins (FLP) as described previously (Lingner et al., 2011)*.

The prediction by *PredPlantPTS1* is presently limited to single sequences. However, PTS1 predictions for multiple sequences and whole genomes can be provided by the authors upon request.

### Ambiguous predictions: Computational validation by PTS1 prediction of putative orthologs

In case of protein sequences carrying non-canonical plant PTS1 tripeptides and ambiguous PTS1 protein prediction scores close to the threshold and posterior probabilities around 50%, the predictions can be often strengthened or falsified by relatively straightforward additional bioinformatic analyses. The underlying concept is the following: if one unknown protein is targeted to peroxisomes by the PTS1 pathway in one plant species, then all its orthologs are generally targeted to peroxisomes by the PTS1 pathway as well (Reumann et al., 2004; Lingner et al., 2012). Hence, by identifying putatively orthologous proteins in the protein database for one specific putative PTS1 protein of interest and analyzing the C-termini of these sequences for the presence of PTS1 tripeptides and PTS1 protein targeting prediction using *PredPlantPTS1*, additional data can often be obtained that further raise the probability for peroxisome targeting.

We applied this approach to two example sequences from different plant species. The first ambiguous protein is a small unknown protein from *Populus trichocarpa* (XP_002313892, 132 aa), which terminates with KVSDEQLALLLIKL> and is given a total PWM prediction score of 0.293 below threshold. The standard posterior probability is 3.7% predicting non-peroxisomal localization, and the balanced posterior probability is 76.4% predicting a PTS1 protein. IKL> had been characterized as a functional but non-canonical PTS1 tripeptides for one *Arabidopsis* protein (At3g44830.1, LCAT, RMSERISIKL>, Table 1) by *in vivo* subcellular targeting analysis (Lingner et al., 2011).

**Figure 3:**
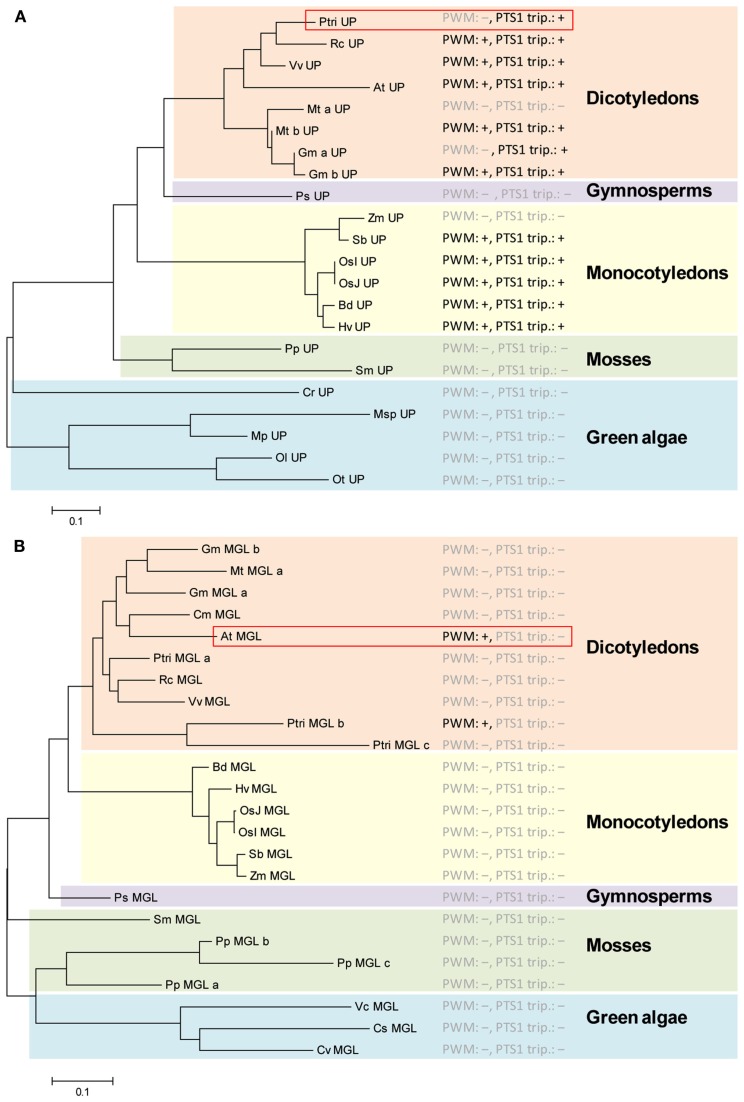
**Analysis of predicted PTS1 conservation in putative orthologs of ambiguously predicted plant PTS1 proteins by a combination of phylogenetic and PTS1 prediction analysis**. Two ambiguously predicted, putative PTS1 proteins from *P. trichocarpa* (XP_002313892) **(A)** and *Arabidopsis thaliana* (NP_176647) **(B)** were blasted against the non-redundant protein database of GenBank. Putatively orthologous proteins (including in-paralogs) were identified in spermatophyta including eudicotyledons (e.g., *Arabidopsis*, *Ricinus*), monocotyledons (*Liliopsida*, *Oryza*, *Zea*), and gymnosperms (*Coniferopsida*, *Picea*), in mosses (*Lyciopodiophyta*, *Selaginella*; Bryophyta, *Physcomitrella*), and in microalgae (chlorophyta, e.g., *Micromonas*, *Ostreococcus*). The sequences were aligned using ClustalX, and the phylogenetic relationship among the sequences was analyzed by the neighbor joining method using MEGA 5. For all putative orthologs the PWM-based PTS1 protein prediction scores and the presence of experimentally validated PTS1 tripeptides were determined (Tables A2 and A3 in Appendix). Positive (+) and negative (−) PWM-based PTS1 protein predictions (e.g., PWM:+) and experimentally validated PTS1 tripeptides (PTS1 trip.:+) are indicated. For At UP (At4g33925) the predictions are given only for the first splice variant.

By a standard BLAST search of the *P. trichocarpa* protein of interest against the protein database of GenBank, a number of homologs can be detected. The query protein shares only marginal sequence similarity with the most closely related homolog in the same species (XP_002303453, 37% identity over 30 aa, *E*-value 0.014), indicating that the protein of interest does not belong to a gene family nor is paralogous to another *P. trichocarpa* protein, which significantly facilitates the detection of orthologous proteins in different plant species. For most plant species, single homologs of similar size and high sequence similarity (dicotyledons: 70–82% identity, *E*-value 10^−77^ to 10^−65^; monocotyledons: 61–68% identity, *E*-value 10^−55^ to 10^−41^) are identified. Phylogenetic analysis by the neighbor joining method further supports the idea that the detected homologs are orthologous to the query protein from *P. trichocarpa* (Figure 3A). Two apparent in-paralogs resulting from gene duplications can be detected for *Medicago* and *Glycine*. The orthologous *Arabidopsis* gene is expressed in two splice variants that differ in their C-termini and PTS1 protein predictions (At4g33925.1, SKI>; At4g33925.2, KCQ>, Table A2 in Appendix).

Except for *Zea mays* (VNL>), at least one putative ortholog of all dicotyledons and monocotyledons included in the analysis terminates with either a non-canonical (SKV>, VKL>) or even a canonical PTS1 tripeptide (SKL>, SKI>, AKL>, Table A2 in Appendix, Figure 3A). Moreover, except for one of two in-paralogs or splice variants, nearly all putative orthologs are predicted PTS1 proteins according to *PredPlantPTS1*. Hence, these bioinformatic data strongly support the hypothesis that the unknown, ambiguously predicted *Populus* protein is indeed targeted to peroxisomes by the PTS1 pathway.

The second ambiguous protein is NP_176647, At1g64660, encoding *Arabidopsis* methionine gamma-lyase (MGL), which catalyzes the first step of Met catabolism (Rebeille et al., 2006; Joshi and Jander, 2009). Even though reported to be cytosolic, At MGL terminates with the PTS1-related tripeptide LRM> and is given a total PWM prediction score of 0.455 above threshold with a standard posterior probability of 74.2% (Table A3 in Appendix, Figure 3B). The protein is encoded by a single gene, and putative orthologs and in-paralogs can be retrieved from the protein database for several plant species (Table A3 in Appendix). However, except for one of three in-paralogs of *P. trichocarpa*, none of the putative orthologs is a predicted PTS1 protein nor carries a functional PTS1 tripeptide (Figure 3B). Hence, these bioinformatic data strongly argue against the prediction that *Arabidopsis* MGL is a PTS1 protein.

### PTS1 protein prediction in mosses and algae

For development of the PWM prediction models we restricted the positive example sequences to spermatophytes because peroxisome targeting is most conserved among orthologs of this plant group. Therefore, the prediction algorithms are most suitable for spermatophytes. However, we noticed that many PTS1 protein orthologs from lower eukaryotes such as mosses (lycopodiophyta, e.g., *Selaginella*; bryophyta, *Physcomitrella*) and green algae (chorophyta) carry canonical or non-canonical PTS1 tripeptides as well, strongly indicating that (i) many PTS1 proteins have been directed to peroxisomes at early stages of evolution of the green lineage and that (ii) the PTS1s are very similar between higher and lower plants. Hence, *PredPlantPTS1* also appears to perform rather well with protein sequences from lower plants, allowing evolutionary analysis of the plant PTS1 proteome.

## Summary

Here we presented *PredPlantPTS1*, an easy-to-use web interface for prediction of plant PTS1 proteins. By means of the underlying prediction algorithm, *PredPlantPTS1* allows the identification of non-canonical and low-abundance PTS1 proteins. The web server provides detailed prediction output including the highlighting of targeting-relevant residues and performs an evaluation of verified PTS1 tripeptides. Future work will comprise the refinement of the prediction model with newly identified PTS1 sequences and the corresponding protein and EST orthologs. Furthermore, we will extend our online resource to prediction of peroxisomal proteins carrying the PTS2 signal and to other taxonomic domains such as animals and fungi.

## Conflict of Interest Statement

The authors declare that the research was conducted in the absence of any commercial or financial relationships that could be construed as a potential conflict of interest.

## Appendix

**Table A1:** **Overview table of experimentally validated plant PTS1 tripeptides**.

AHL>	FKL>	SFM>	SNL>	SSI>
AKI>	GRL>	SGL>	SNM>	SSL>
AKL>	IKL>	SHI>	SPL>	SSM>
ALL>	KRL>	SKI>	SQL>	STI>
ANL>	LKL>	SKL>	SRF>	STL>
ARL>	PKI>	SKM>	SRI>	SYM>
ARM>	PKL>	SKV>	SRL>	TRL>
ASL>	PRL>	SLL>	SRM>	VKL>
CKI>	SCL>	SLM>	SRV>	
CKL>	SEL>	SML>	SRY>	

**Table A2:** **Strengthening of PTS1 protein prediction for an ambiguously predicted *Populus* protein by ortholog analysis**.

Accession	Species	Annotation	Group	C-term. 14 aa	PWM score	Post. prob.(%)	Pred.	Exp. PTS1 tripeptide validation
XP**_**002313892	*Populus trichocarpa*	Predicted protein	Eudicotyledons		0.293	3.7	C	Val.
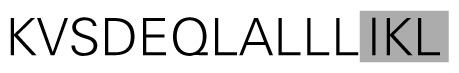
At4g33925.1	*Arabidopsis thaliana*	Uncharacterized protein	Eudicotyledons		0.789	99.9	P	Val.
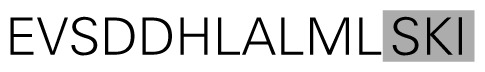
At4g33925.2	*Arabidopsis thaliana*	Uncharacterized protein	Eudicotyledons	SLGTYNEIEAYKCQ	−1.178	0	C	Not val.
XP_002518659	*Ricinus communis*	Conserved hypothetical protein	Eudicotyledons		0.469	80.2	P	Val.
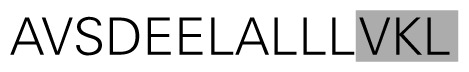
XP_002272459	*Vitis vinifera*	Zinc finger SWIM domain-containing protein 7	Eudicotyledons		0.965	100.0	P	Val.
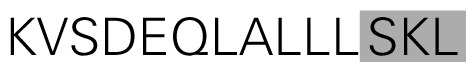
XP_003527578	*Glycine max*	Zinc finger SWIM domain-containing protein 7-like	Eudicotyledons		0.198	0.2	C	Val.
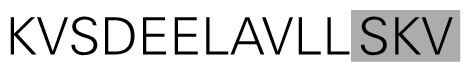
XP_003523843	*Glycine max*	Zinc finger SWIM domain-containing protein 7-like	Eudicotyledons		0.584	98.1	P	Val.
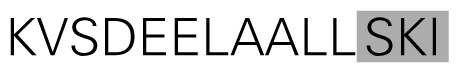
XP_003598325	*Medicago truncatula*	Zinc finger SWIM domain-containing protein	Eudicotyledons		0.675	99.7	P	Val.
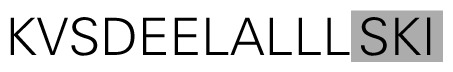
XP_003605702	*Medicago truncatula*	Zinc finger SWIM domain-containing protein	Eudicotyledons	EVKVSDEELAFVAI	−0.603	0.0	C	Not val.
EEC82375	*Oryza sativa Ind*.	Hypothetical protein OsI_26711	Liliopsida		0.901	100.0	P	Val.
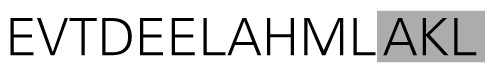
NP_001060165	*Oryza sativa Jap*.	Os07g0593200 (partial)	Liliopsida		0.901	100.0	P	Val.
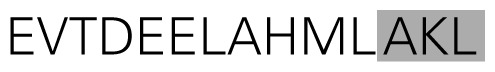
NP_001144742	*Zea mays*	Uncharacterized protein LOC100277790	Liliopsida	EVKDEELANMLVNL	−0.069	0.0	C	Not val.
XP_003560014	*Brachypodium distachyon*	Zinc finger SWIM domain-containing protein 7-like	Liliopsida		0.901	100.0	P	Val.
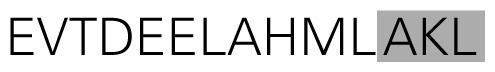
BAK05023	*Hordeum vulgare*	Predicted protein	Liliopsida		0.901	100.0	P	Val.
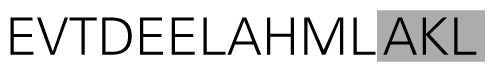
XP_002463119	*Sorghum bicolor*	Hypothetical protein SORBIDRAFT_02g038190	Liliopsida		0.810	100.0	P	Val.
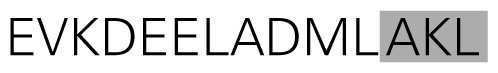
ABR17386	*Picea sitchensis*	Unknown	Coniferopsida	KISDEQLALLLLKH	−0.607	0.0	C	Not val.
XP_001767328	*Physcomitrella patens*	Predicted protein	Bryophyta	TVSDAELAHLLLQC	−1.054	0.0	C	Not val.
XP_002988124	*Selaginella moellendorffii*	Hypothetical protein SELMODRAFT_127426, partial	Lycopodiophyta	KRNRRQVHYALQEK	−1.233	0.0	C	Not val.
XP_002503713	*Micromonas* sp.	Predicted protein	Chlorophyta	SRRGGGGGGNGGRR	−0.817	0.0	C	Not val.
XP_003059293	*Micromonas pusilla*	Predicted protein	Chlorophyta	GGGGGGRQPPPTFR	−1.131	0.0	C	Not val.
XP_001417589	*Ostreococcus lucimarinus*	Predicted protein	Chlorophyta	LGNMMMKSFEDDPM	−0.835	0.0	C	Not val.
XP_003078967	*Ostreococcus tauri*	Unnamed protein product	Chlorophyta	GNMMMKYFEGDAAM	−0.142	0.0	C	Not val.
XP_001692127	*Chlamydomonas reinhardtii*	Hypothetical protein (partial)	Chlorophyta	PDYTIAHMLLEHCA	−1.156	0.0	C	Not val.

*An ambiguously predicted, putative PTS1 protein from *Populus trichocarpa* protein (XP_002313892) was blasted against the non-redundant protein database of GenBank. Putatively orthologous proteins (including in-paralogs) were identified in spermatophyta including eudicotyledons (e.g., *Arabidopsis*, *Ricinus*), monocotyledons (*Liliopsida*, *Oryza*, *Zea*), and gymnosperms (*Coniferopsida*, *Picea*), in mosses (lycopodiophyta, *Selaginella*; Bryophyta, *Physcomitrella*), and in microalgae (chlorophyta, e.g., *Micromonas*, *Ostreococcus*). For all protein sequences PWM-based prediction scores were determined by *PredPlantPTS1*. PTS1 protein predictions and experimentally validated PTS1 tripeptides are shaded in gray*.

**Table A3:** **Falsifying PTS1 protein prediction for the ambiguously predicted *Arabidopsis* methionine gamma lyase by ortholog analysis**.

Accession	Species	Annotation	Group	C-term. 14 aa	PWM score	Post. prob.(%)	Pred.	Exp. PTS1 tripeptide validation
At1g64660	*Arabidopsis thaliana*	Methionine gamma-lyase	Eudicotyledons	EQKWTQFEKAFLRM	0.455	74.2	P	not val.
XP_002299428	*Populus trichocarpa*	Predicted protein	Eudicotyledons	KAFSRLQDSGLYKN	−0.651	0	C	not val.
XP_002304835	*Populus trichocarpa*	Predicted protein	Eudicotyledons	EQKWSQFTKAYSEM	0.469	80.0	P	not val.
XP_002336096	*Populus trichocarpa*	Predicted protein	Eudicotyledons	KWNQFKSAYEEMKE	−0.350	0	C	not val.
XP_002518910	*Ricinus communis*	Cystathionine gamma-synthase, putative	Eudicotyledons	SQFEKALSRMKECY	−1.149	0	C	not val.
XP_002280162	*Vitis vinifera*	Methionine gamma-lyase-like	Eudicotyledons	RWSQFEKALSRMQG	−0.724	0	C	not val.
ADN33936	*Cucumis melo* subsp. *melo*	Cystathionine gamma-synthase	Eudicotyledons	LAKVQDIGVPFCNN	−0.669	0	C	not val.
XP_003536171	*Glycine max*	Methionine gamma-lyase-like	Eudicotyledons	ALTRLNDSGYNKIA	−1.271	0	C	not val.
XP_003520012	*Glycine max*	Methionine gamma-lyase-like	Eudicotyledons	EMALERFQEKEPLV	−0.992	0	C	not val.
XP_003601451	*Medicago truncatula*	Cystathionine gamma-lyase	Eudicotyledons	SQLEKAVIKFNEKH	−0.716	0	C	not val.
EAY79213	*Oryza sativa Ind*.	Hypothetical protein OsI_34329	Liliopsida	DAAAKYCKIVEWHS	−1.181	0	C	not val.
NP_001065069	*Oryza sativa Jap*.	Os10g0517500	Liliopsida	QHPDRDAAAKYCKV	−0.132	0	C	not val.
NP_001152224	*Zea mays*	O-succinylhomoserine sulfhydrylase	Liliopsida	DRDGPEAANNHRKH	−0.523	0	C	not val.
XP_003574196	*Brachypodium distachyon*	Cystathionine gamma-lyase-like	Liliopsida	QDAPSAAAKYCKAI	−0.871	0	C	not val.
BAK03127	*Hordeum vulgare*	Predicted protein	Liliopsida	TPAAAATAKYGKAV	−1.281	0	C	not val.
XP_002464368	*Sorghum bicolor*	Hypothetical protein	Liliopsida	RDGSDAAGNNHRKH	−0.587	0	C	not val.
ABK27101	*Picea sitchensis*	Unknown	Coniferopsida	ALTSMTEVLPSKRM	0.286	3.1	C	not val.
XP_001751901	*Physcomitrella patens*	Predicted protein	Bryophyta	TSLKLVPDSAKWLD	−1.166	0	C	not val.
XP_001759514	*Physcomitrella patens*	Predicted protein	Bryophyta	LKLVPPQAVDSSVR	−0.710	0	C	not val.
XP_001756897	*Physcomitrella patens*	Predicted protein	Bryophyta	DLVAHNLIPSLTVD	−1.135	0	C	not val.
XP_002961730	*Selaginella moellendorffii*	Hypothetical protein	Lycopodiophyta	LHDAIVALGIARKA	−0.523	0	C	not val.
EIE26481	*Coccomyxa ellipsoidea C-169*	Cystathionine gamma-synthase	Chlorophyta	YRAAEVRPDPFPSS	−0.538	0	C	not val.
XP_002955875	*Volvox carteri f. nagariensis*	Hypothetical protein	Chlorophyta	RWRQLEEAYRFVMQ	−0.819	0	C	not val.
EFN56203	*Chlorella variabilis*	Hypothetical protein	Chlorophyta	SAEHSKDAIAATAK	−1.166	0	C	not val.

*An ambiguously predicted, putative PTS1 protein from *Arabidopsis thaliana* (At1g64660) was blasted against the non-redundant protein database of GenBank. Putatively orthologous proteins (including in-paralogs) were identified in spermatophyta including eudicotyledons (e.g., *Arabidopsis*, *Ricinus*), monocotyledons (*Liliopsida*, *Oryza*, *Zea*), and gymnosperms (*Coniferopsida*, *Picea*), in mosses (*Lycopodiophyta*, *Selaginella*; bryophyta, *Physcomitrella*), and in microalgae (chlorophyta, e.g., *Micromonas*, *Ostreococcus*). For all protein sequences PWM-based prediction scores were determined by *PredPlantPTS1*. PTS1 protein predictions and‘ experimentally validated PTS1 tripeptides are shaded gray*.
